# Cryo-EM of kinesin-binding protein: challenges and opportunities from protein-surface interactions

**DOI:** 10.1107/S2059798321001935

**Published:** 2021-03-30

**Authors:** Joseph Atherton, Carolyn A. Moores

**Affiliations:** a Randall Centre for Cell and Molecular Biophysics, King’s College London, London, United Kingdom; b Institute of Structural and Molecular Biology, Birkbeck, University of London, London, United Kingdom

**Keywords:** cryo-EM, sample preparation, graphene oxide, graphene, kinesin-binding protein

## Abstract

Kinesin-binding protein (KBP) is an important selective inhibitor of specific kinesin family members and its genetic disruption causes Goldberg–Shprintzen syndrome. Cryo-electron microscopy (cryo-EM) has recently been used to reveal the structure of KBP alone (72 kDa) and in complex with the motor domain of the mitotic kinesin-12 KIF15 (110 kDa). KBP is an α-solenoid, tetratricopeptide-repeat protein that interacts with the microtubule-binding region of the kinesin motor domain and blocks microtubule attachment. Numerous challenges arose relating to the behavior of KBP and KBP–kinesin complexes during cryo-EM sample preparation. These included the partial denaturation of KBP by air–water interfaces, protein aggregation resulting from carbon interaction and preferential orientation. Sample preparation with a graphene oxide substrate enabled the eventual structure determination. Here, experiences with preparing these samples are detailed, bringing attention to some of the challenges and opportunities that are likely to arise from protein-surface interactions.

## Abstract 2

Sample preparation is a major limiting factor in cryo-electron microscopy (cryo-EM), particularly for small macromolecules under 100 kDa. Here, experiences in preparing kinesin-binding protein (KBP; 72 kDa) and KBP–kinesin motor domain (110 kDa) samples for cryo-EM are detailed, in particular some of the challenges and opportunities arising from surface interactions on the cryo-EM grid.

## 1. Introduction   

Since the recent revolution in hardware and software (Kühlbrandt, 2014 ▸), cryo-electron microscopy (cryo-EM) has become a popular and effective method of macromolecular structure determination. With imaging and processing technology now enabling atomic resolution (Nakane *et al.*, 2020 ▸; Yip *et al.*, 2020 ▸), the preparation of suitable samples remains a major limiting factor. In recent years, there has been a growing awareness that macromolecules interact with various surfaces on EM grids during sample preparation and that this can cause protein unfolding and/or conformational artifacts (Glaeser & Han, 2017 ▸).

In this technical report, we detail our experiences when preparing samples of kinesin-binding protein (KBP; 72 kDa) alone or in complex with two different kinesin motor domains (∼40 kDa). KBP is important in a number of cellular processes, including neuronal development, spermatogenesis and mitosis, and its mutation causes Goldberg–Shprintzen syndrome (GOSHS; Alves *et al.*, 2010 ▸; Brooks *et al.*, 2005 ▸; Dafsari *et al.*, 2015 ▸; Drerup *et al.*, 2016 ▸; Lehti *et al.*, 2013 ▸; Lyons *et al.*, 2008 ▸; Malaby *et al.*, 2019 ▸; Salehpour *et al.*, 2017 ▸; Valence *et al.*, 2013 ▸). KBP functions as a selective inhibitor of microtubule attachment of a subset of kinesin motor proteins (Kevenaar *et al.*, 2016 ▸; Wozniak *et al.*, 2005 ▸), and our recent structural work describes the inhibitory mechanism of KBP (Atherton *et al.*, 2020 ▸). Here, we describe a number of effects on our samples derived from protein-surface interactions during cryo-EM sample preparation that we hope will be informative to other researchers in the field: (i) partial denaturation of KBP, likely due to interactions with the air–water interface, (ii) the protection of KBP from partial denaturation by adherence to a graphene oxide (GO) substrate away from the air–water interface, (iii) changes to KBP angular distributions introduced by adherence to GO, (iv) aggregation in KBP–kinesin motor domain (MD) complexes resulting from interactions with a carbon grid support, which was not observed on a gold grid support, and (v) different behaviors of two KBP–kinesin motor domain (MD) complexes on a GO substrate. In addition, we also highlight technical issues with the use of GO, and with combining Volta phase plate (VPP) cryo-EM with tilted data collection.

## 2. Methods   

### 2.1. Protein expression and purification for cryo-EM   

Full-length human KBP (residues 1–621 in a PSTCm1 expression vector with kanamycin resistance and an N-terminal thrombin-cleavable 6×His tag) was expressed in *Escherichia coli* Rosetta2 cells (Novagen) and purified using immobilized metal-affinity chromatography (IMAC) and size-exclusion chromatography (SEC) as described previously (Atherton *et al.*, 2020 ▸; Kevenaar *et al.*, 2016 ▸).

A human KIF1A motor domain and neck linker construct (KIF1A_MD; residues 1–362) in a pFN18a vector (with a TEV protease-cleavable N-terminal HaloTag and a C-terminal 6×His tag) was expressed in *E. coli* BL21-Gold (DE3) cells and purified via IMAC and SEC as described previously (Atherton *et al.*, 2014 ▸, 2020 ▸). A cysteine-light human KIF15 motor domain and neck linker construct (residues 1–375; referred to as KIF15_MD6S as six of its eight cysteine residues were mutated to serines, C5S, C50S, C162S, C294S, C314S and C346S, and two additional cysteines were inserted, S250C and G375C) in a pET-21a vector with a C-terminal 6×His tag was expressed and purified as described previously (Rosenfeld *et al.*, 2005 ▸).

KBP–KIF15_MD6S or KBP–KIF1A_MD complexes were purified via IMAC using the 6×His tag on the kinesin constructs after incubation with a tenfold excess of KBP, as described previously (Atherton *et al.*, 2020 ▸).

### 2.2. Sample preparation for cryo-EM   

For KBP sample preparation, KBP stored in 20 m*M* Tris–HCl pH 7.4, 150 m*M* NaCl, 2.5 m*M* CaCl_2_, 1 m*M* DTT was diluted with KBP dilution buffer (20 m*M* Tris–HCl pH 7.5, 150 m*M* NaCl, 2 m*M* DTT) to either 0.15, 0.3 or 0.02 mg ml^−1^ for application onto glow-discharged C-flat 2/2 holey carbon EM grids (Protochips, Morrisville, North Carolina, USA), 1.2/1.3 UltrAuFoil gold EM grids (Quantifoil) or C-flat 2/2 holey carbon EM grids with overlaid GO (Sigma), respectively.

For the preparation of kinesin MD–KBP complexes on GO-coated gold grids, samples were diluted with KBP dilution buffer plus 0.2 m*M* ADP to 0.03 mg ml^−1^ and added to glow-discharged 1.2/1.3 UltrAuFoil gold EM grids (Quantifoil) with overlaid GO. For the preparation of kinesin MD–KBP complexes on GO-free gold grids, samples were diluted with KBP dilution buffer plus 0.2 m*M* ADP to 0.15 mg ml^−1^ and applied onto glow-discharged 1.2/1.3 UltrAuFoil gold EM grids (Quantifoil).

4 µl of each sample was added to EM grids and after a 30 s incubation in a Vitrobot Mark IV (FEI, Hillsboro, Oregon, USA) at 4°C and 80% humidity, samples were blotted (6–8 s, blot force −10) and vitrified in liquid ethane. GO pre-coating of grids was performed according to the protocol described by Cheng *et al.* (2020 ▸). All steps were performed at 4°C.

### 2.3. Cryo-EM data collection   

With the exception of Fig. 6(*b*) and Supplementary Fig. S4, all data were collected on Titan Krios electron microscopes (Thermo Fisher) operating at 300 kV with a K2 Summit direct electron detector (Gatan, California, USA) and a Quantum post-column energy filter (Gatan) operated in zero-loss imaging mode. Low-dose movies were collected automatically using *EPU* (Thermo Fisher, Massachusetts, USA).

Data sets for KBP alone additionally used a Volta phase plate (VPP), a sampling of ∼1.05 Å per pixel and a total dose of 42 e^−^ Å^−2^ over 40 frames, with the detector operating in counting mode at a rate of ∼5 e^−^ per pixel per second. Untilted data sets had a nominal defocus range of 0.5–0.7 µm, whilst a data set collected at a 40° stage tilt had a nominal defocus range of 0.5–1.2 µm. Data sets for KBP–kinesin complexes were collected without a VPP with a nominal defocus range of 1.5–4 µm. KBP–KIF1A_MD complexes were collected at a sampling of 0.85 Å per pixel, whereas KBP–KIF15_MD6S complexes were collected at a sampling of 1.047 Å per pixel. For KBP–KIF1A_MD complexes the total dose was 88 e^−^ Å^−2^ over 36 frames, with the detector operating in counting mode at a rate of 7.1 e^−^ per pixel per second. For KBP–KIF15_MD6S complexes, the total dose was 80 e^−^ Å^−2^ over 64 frames, with the detector operating in counting mode at a rate of 5.7 e^−^ per pixel per second.

The screening images shown in Fig. 6(*b*) were collected manually at the ISMB, Birkbeck on a Tecnai T12 microscope (Thermo Fisher) operating at 120 kV using a CCD camera (Gatan). The small data set shown in Supplementary Fig. S4 was collected at the ISMB, Birkbeck on a Tecnai G2 Polara microscope (Thermo Fisher) operating at 300 kV with a K2 Summit direct electron detector (Gatan) and a Quantum post-column energy filter (Gatan) operated in zero-loss imaging mode. A VPP was not used and a nominal defocus range of 1.5–4 µm and a final pixel size of 1.39 Å were used. The total dose was 42 e^−^ Å^−2^ over 64 frames at a counting-mode rate of 5.1 e^−^ per pixel per second.

### 2.4. Cryo-EM data processing and model building   

Cryo-EM data were processed as described previously (Atherton *et al.*, 2020 ▸). Briefly, low-dose dose-weighted movies were motion-corrected using *MotionCor*2 (Zheng *et al.*, 2017 ▸) and CTF determination was performed with *Gctf* (Zhang, 2016 ▸). Particles were picked from good micrographs using the neural network picker in *EMAN*2 (Bell *et al.*, 2018 ▸) and *Gautomatch* (http://www.mrc-lmb.cam.ac.uk/kzhang/). Particles were initially processed with *cryoSPARC*2 (Punjani *et al.*, 2017 ▸), *cisTEM* (Grant *et al.*, 2018 ▸) and *RELION* version 3.0 (Zivanov *et al.*, 2018 ▸) before data combination, duplicate removal and final processing in *RELION* version 3.1 (Zivanov *et al.*, 2018 ▸). 3D reconstructions were sharpened with negative *B* factors applied to the gold-standard FSC 0.143 cutoff as described previously (Atherton *et al.*, 2020 ▸).

All displayed 2D classes are displayed in *RELION*, while 3D molecular representations were made using the *UCSF Chimera* or *ChimeraX* software (Goddard *et al.*, 2018 ▸; Pettersen *et al.*, 2004 ▸). 3D molecular models were built with *MODELLER* (Šali & Blundell, 1993 ▸), *Flex-EM* (Topf *et al.*, 2008 ▸) and *Coot* (Emsley *et al.*, 2010 ▸) based on a combination of secondary-structure prediction, TPR prediction, fragment homology information and prior knowledge of right-handed α-solenoid proteins, guided by the cryo-EM density, and were then refined with *Phenix* (Liebschner *et al.*, 2019 ▸) as described previously (Atherton *et al.*, 2020 ▸). Data-collection and model-refinement statistics can be found in our previous publication (Atherton *et al.*, 2020 ▸).

### 2.5. Data availability   

The final densities and fitted models were deposited in the Electron Microscopy Data Bank (EMDB) and Protein Data Bank (PDB), respectively, following our previous publication (Atherton *et al.*, 2020 ▸) with the following codes: KBP, EMDB entry EMD-11338, PDB entry 6zpg; KBP–KIF15_MD6S, EMDB entry EMD-11339, PDB entry 6zph.

## 3. Results   

### 3.1. KBP becomes partially denatured at the air–water interface, a process prevented by adherence to a graphene oxide substrate   

**Figure 1:**
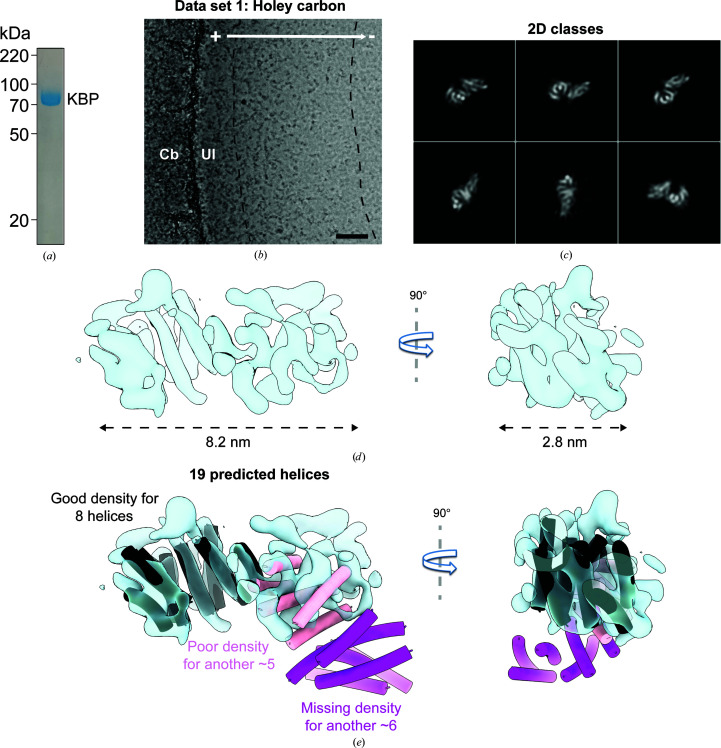
KBP sample preparation on holey carbon grids reveals a partially denatured species. (*a*) SDS–PAGE gel of purified KBP (∼72 kDa). (*b*) Representative micrograph of KBP prepared on holey carbon grids, showing a gradient of ice thickness and protein concentration in holes in the carbon (+ to −, high to low ice thickness). The transition region between thicker and thinner ice that contained particles contributing to good 2D classes is found roughly between the black dashed lines. Cb, carbon support; UI, unsupported ice. Scale bar = 40 nm. (*c*) Exemplar well populated 2D classes of KBP prepared on holey carbon grids. (*d*) 3D reconstruction of KBP prepared on holey carbon grids. Experimental density is shown in semi-transparent light blue. (*e*) The same as (*d*) but with our final published KBP model (PDB entry 6zpg), containing 19 helices arranged in a right-handed α-solenoid, shown fitted to the density; eight helices fit well to part of the density (black tubes), five fit poorly (pink tubes) and there is no density for the remaining six (magenta tubes). 90° rotated views are shown as indicated.

Following recombinant *E. coli* expression and purification of human KBP (72 kDa; Fig. 1 ▸ *a*), our first sample-preparation attempt for cryo-EM used copper grids with an overlying holey carbon film (see Section 2). On such grids the sample is imaged in the holes in the carbon film that contain unsupported vitreous ice. Samples were screened at 120 kV on a Tecnai T12 without a phase plate and with a CCD camera. Although particle distribution can be observed in thin ice regions and therefore some aspects of sample preparation could be optimized, this screening setup is not suitable to generate images for 2D classification or for the determination of the orientations of such small particles. Next, data were collected at 300 kV on a Titan Krios with a K2 camera and a Volta phase plate (Fig. 1 ▸ *b*), which we felt would be beneficial for a particle under 100 kDa when at this operating voltage. Due to the small particle size, we picked particles from grid regions with the thinnest possible ice: both the ice thickness and the particle concentration decreased dramatically away from the carbon edges towards the center of the holes (Fig. 1 ▸ *b*). Particles were excluded from the thinnest ice regions at the center of the holes, while particles were overcrowded in the thicker ice regions closer to the carbon edge. A subset of selected particles, predominantly from the transition region between thinner and thicker hole ice, contributed to well populated good 2D classes where secondary structure could be seen (Fig. 1 ▸ *c*). Processing these good 2D classes allowed the calculation of a *de novo* 3D reference, and subsequent 3D refinement produced a subnanometre-resolution 3D structure of ∼8 nm in length and 3 nm in width (Fig. 1 ▸ *d*). Secondary-structure prediction suggested that KBP is built from 19 helices, but clear ‘sausage-like’ density for only eight helices was observed. Ultimately, the KBP structure was indeed found to have 19 helices as predicted, arranged in a right-handed α-solenoid (Atherton *et al.*, 2020 ▸). When this final structure was fitted into the KBP density from unsupported ice, eight helices were a good fit, another five fitted poorly and there was missing density for another six (Fig. 1 ▸ *e*).

**Figure 2:**
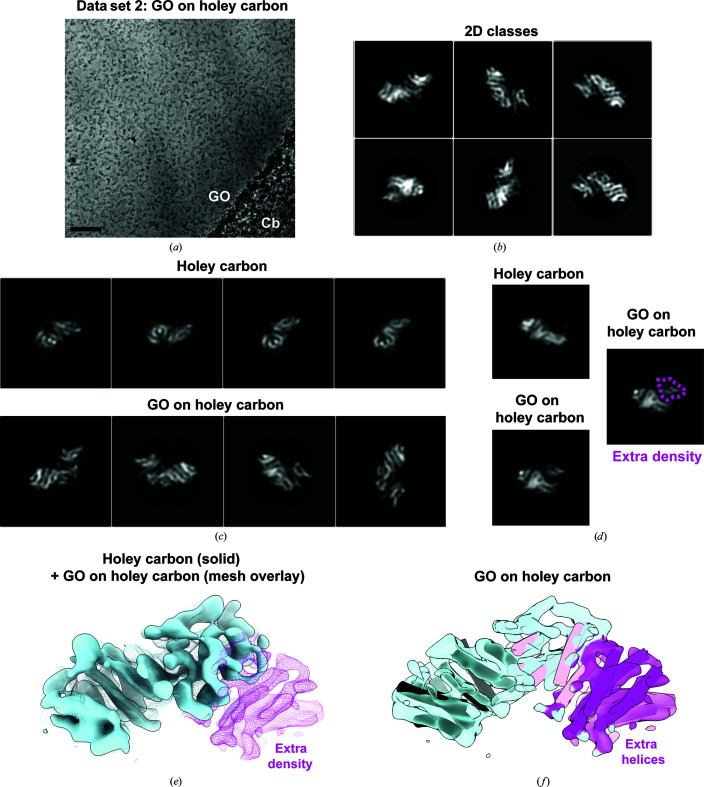
Adherence of KBP to a GO substrate prevents partial denaturation. (*a*) Representative micrograph of KBP prepared on holey carbon grids coated with GO. GO, graphene oxide layered over holes in the carbon. Cb, carbon support. Scale bar = 40 nm. (*b*) Exemplar well populated 2D classes of KBP prepared on holey carbon grids coated with GO. (*c*) Comparison of well populated 2D classes of KBP prepared on holey carbon grids with or without a GO coating. (*d*) Selected 2D classes from data on holey carbon or GO on holey carbon (left top and bottom panels, respectively). These 2D classes appeared to be similar views of KBP, except with extra density in the 2D class on GO, as indicated with a dashed magenta line in the right-hand panel. (*e*) 3D reconstruction calculated from well populated KBP 2D classes from holey carbon grids (solid light blue density) superimposed onto a 3D reconstruction of well populated KBP 2D classes from GO-coated holey carbon grids (mesh). The magenta-colored mesh indicates extra density in the reconstruction from GO-coated holey carbon grids. (*f*) 3D reconstruction of well populated KBP 2D classes from GO-coated holey carbon grids. Semi-transparent magenta density indicates extra density as in (*e*). Modeled predicted helices are fitted (tubes), with all 19 now accounted for, including six helices that were only apparent in the reconstruction from well populated KBP 2D classes from GO-coated holey carbon grids (magenta tubes; compare with Fig. 1 ▸ *d*).

At the time, the discrepancy between the predicted structure and the experimental density led us to speculate that part of the protein was becoming denatured during sample preparation, possibly upon interaction with the air–water interface. In order to examine this possibility, we prepared the sample on the same grids but with a pre-applied GO substrate and collected data under identical conditions (Fig. 2 ▸ *a*), with the idea that particles would potentially adhere to the GO substrate and would therefore be sequestered away from the air–water interface. Approximately tenfold lower sample concentrations were used with a GO substrate compared with without a GO substrate, in order to achieve similar particle-distribution densities (see Section 2). However, the particle distribution and sample quality varied substantially in different areas of the grids: while some regions had excellent particle distribution, other regions lacked GO coverage and particles, whereas protein aggregation or particle overcrowding was observed in other regions, with GO overlaps, folds or multiple layering (Supplementary Fig. S1). Processing of particles from regions with good particle distribution (not overcrowded or aggregated) led to 2D classes with clear secondary structure (Fig. 2 ▸ *b*). Strikingly, 2D classes derived from particles on GO appeared to be larger than those derived from particles without a GO substrate (Fig. 2 ▸ *c*). Comparison of selected 2D views illustrated clear extra density in the 2D classes derived from particles on GO (Fig. 2 ▸ *d*). Generation of a *de novo* 3D reference from, and the subsequent alignment of, particles from the GO data set led to a subnanometre-resolution reconstruction of a larger structure with extra density accounting for all 19 predicted helices in KBP (Figs. 2 ▸ *e* and 2 ▸ *f*). We will refer to this species as ‘KBP-full’, while the smaller species missing six helices will be referred to as ‘KBP-partial’.

### 3.2. Adherence of KBP to a graphene oxide substrate results in preferential orientations   

**Figure 3:**
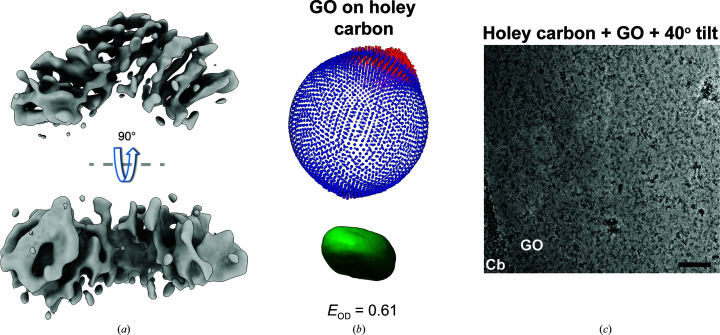
KBP adopts preferred orientations on a GO substrate. (*a*) 90° rotated views of the 3D reconstruction of well populated KBP 2D classes from GO-coated holey carbon grids, illustrating anisotropic smearing. (*b*) 3D angular distribution (top, *RELION* output plot), Fourier space point-spread function (PSF) and efficiency *E*_od_ measure corresponding to the reconstruction and orientation shown in the bottom image in (*a*). The more circular the PSF is, the more isotropic the data are. *E*_od_ is a statistical measure characterizing the angular distribution, where a value of 1 is a perfectly isotropic angular distribution and a value of ∼0.6 is suboptimal (Naydenova & Russo, 2017 ▸). (*c*) Representative micrograph of KBP prepared on holey carbon grids coated with GO with 40° stage tilt applied. GO, graphene oxide layered over holes in the carbon. Cb, carbon support. Scale bar = 40 nm.

While the use of GO allowed us to visualize KBP-full, the reconstruction suffered from anisotropic smearing (Fig. 3 ▸ *a*) due to strong preferential orientations (Fig. 3 ▸ *b*, Table 1 ▸). In an attempt to improve the angular distribution, we collected a data set from the sample on GO under similar conditions, except for the application of a 40° stage tilt (Fig. 3 ▸ *c*). Standard global CTF determination (the determination of one set of values across the whole tilted image) gave inaccurate CTF parameters, as judged by the lack of expected phase-shift progressions at different VPP positions over time, and further particle processing gave only poor-quality 2D classes (Supplementary Fig. S2*a*).

We did not find a program that could accurately determine local CTF parameters across the whole tilted image with phase shifts introduced by a VPP. Therefore, we used custom scripts to determine the CTF in strips along the tilt axis using *Gctf* (Supplementary Fig. S2*b*). This method determined the expected defocus gradient and stable phase shift perpendicular to the tilt axis within each tilted image (Supplementary Fig. S2*b*) in addition to anticipated phase-shift progressions over time at different phase-plate positions (Supplementary Fig. 2*c*), consistent with accurate CTF determination. Despite this, the 2D classes were still of relatively poor quality and 3D reconstruction was not possible (Supplementary Fig. 2*c*, Table 1 ▸), possibly due to the decreased signal to noise introduced by increased sample thickness, charging and beam-induced motion at high specimen tilts (Brilot *et al.*, 2012 ▸; Tan *et al.*, 2017 ▸).

### 3.3. Reprocessing of GO-free data reveals a small number of fully ordered KBP particles giving additional views   

**Figure 4:**
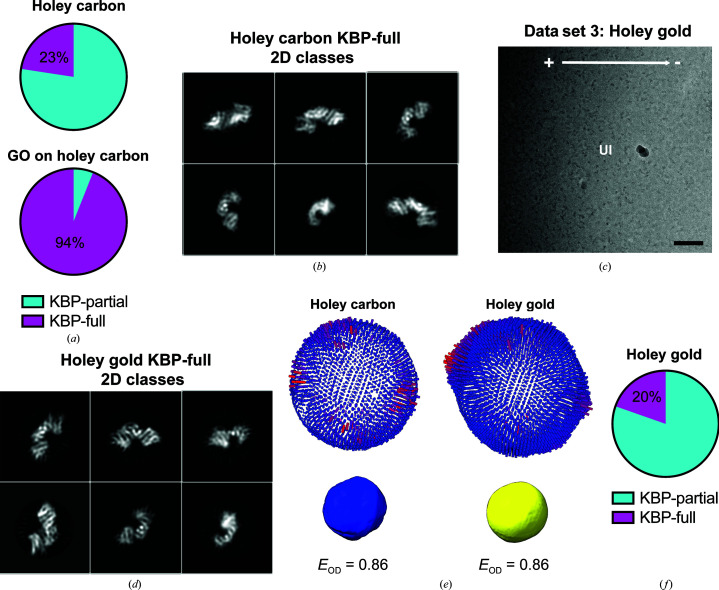
A minority of KBP particles on GO-free holey grids are fully ordered and provide additional 2D views. (*a*) Pie charts indicating the proportion of particles representing KBP-partial or KBP-full species on holey carbon grids or holey carbon grids with a GO substrate. (*b*) Example rare 2D classes of KBP-full prepared on holey carbon grids. (*c*) Representative micrograph of KBP prepared on holey gold grids, showing a gradient of ice thickness and protein concentration in holes in the gold (+ to −, high to low ice thickness). UI, unsupported ice. Scale bar = 40 nm. (*d*) Example rare 2D classes of KBP-full prepared on holey gold grids. (*e*) 3D angular distributions (top, *RELION* output plot) and Fourier PSFs and *E*_od_ (below) for rare KBP-full particles derived from holey carbon (left) and holey gold (right) grids. Orientations of the 3D angular distributions and Fourier PSFs correspond to the KBP reconstruction orientation shown in the bottom image of Fig. 3 ▸(*a*). An *E*_od_ of >0.8 indicates a very good angular distribution (Naydenova & Russo, 2017 ▸). (*e*) Pie chart indicating the proportion of particles representing KBP-partial or KBP-full species on holey gold grids without GO.

We then reanalysed the initial data set from unsupported ice (without a GO surface) to assess the possibility that a minority of particles existed as the KBP-full species but had been missed in our initial analysis. To investigate this, we reprocessed this data set using a larger number of 2D classes and removing the well populated KBP-partial 2D classes. This painstaking reprocessing revealed good 2D classes (secondary-structure elements resolved) of KBP-full, representing roughly 23% of particles in this data set (Figs. 4 ▸ *a* and 4 ▸ *b*, Table 1 ▸). We tested whether the use of holey gold EM grids (UltrAuFoil, see Section 2) without a GO coating could increase the proportion of KBP-full particles by collecting a further data set under similar imaging conditions (without tilting and with a VPP; Fig. 4 ▸ *c*). While this generated further good KBP-full 2D classes and particles, the angular distribution and the ratio of KBP-full to KBP-partial particles were similar to holey carbon EM grids without GO (Figs. 4 ▸ *d*–4 ▸ *f*, Table 1 ▸). Attempts to separate KBP-full and KBP-partial particles with 3D classification approaches was inaccurate compared with the 2D approach and was not pursued.

**Figure 5:**
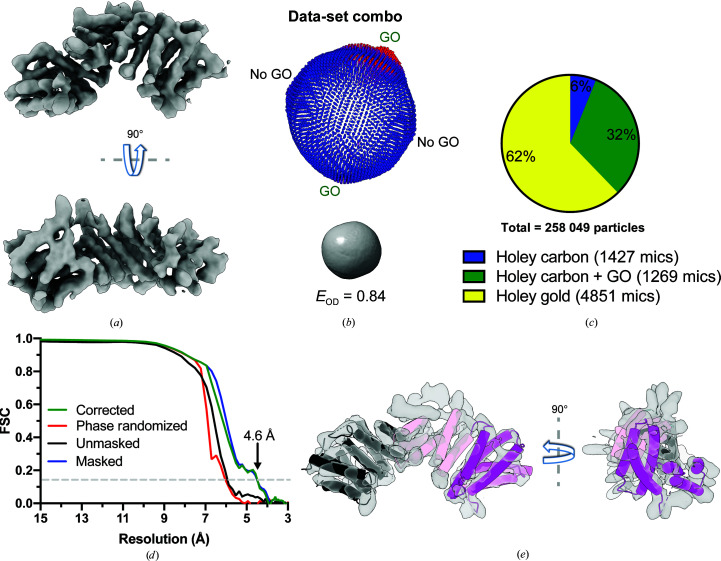
Combining KBP particles from GO-coated and GO-free grids allows the calculation of an isotropic 4.6 Å resolution reconstruction. (*a*) 90° rotated views of the 3D reconstruction of combined KBP-full 2D classes derived from both GO-coated and noncoated holey EM grids, exhibiting isotropic density. (*b*) 3D angular distribution (top, *RELION* output plot) and Fourier PSFs and *E*_od_ (below) for combined KBP-full particles derived from both GO-coated and noncoated (No GO) holey EM grids. The orientation of the 3D angular distribution and Fourier PSF correspond to the KBP reconstruction orientation shown at the bottom of (*a*) (and at the bottom of Fig. 3 ▸ *a*). An *E*_od_ of >0.8 indicates a very good angular distribution (Naydenova & Russo, 2017 ▸). (*c*) Pie chart indicating the proportion of KBP-full particles contributing to the reconstruction in (*a*) from holey carbon, holey gold or holey carbon plus GO grids. (*d*) Gold-standard Fourier shell correlation (FSC) curves between independent masked, unmasked, phase-randomized and corrected half-maps (Chen *et al.*, 2013 ▸) for the combined KBP-full data-set reconstruction shown in (*a*) calculated by *RELION* version 3.1 (Zivanov *et al.*, 2018 ▸; 4.6 Å resolution at the ‘gold-standard’ 0.143 FSC cutoff). (*e*) 90° rotated views of the KBP-full 3D reconstruction shown in (*a*) but with semi-transparent density and helices (as tubes) and loops of the fitted KBP model shown. Tube helices and their respective connecting loops are colored as in Figs. 1 ▸(*d*) and 2 ▸(*f*). Density for the magenta helices is absent in the KBP-partial species.

KBP-full particles isolated in good 2D classes were combined from data sets derived from grids both with and without GO. This combination provided complementary Euler angles to give an overall excellent angular distribution, resulting in an isotropic reconstruction at 4.6 Å resolution (Figs. 5 ▸ *a*–5 ▸ *d*). Although KBP-full particles were a minority in the holey gold data set (without GO; Fig. 4 ▸ *f*), they contributed 62% of the KBP-full particles used in the final reconstruction from combined data sets (Fig. 5 ▸ *c*). As this data set was larger, the sample quality was more consistent and a higher proportion of each hole was usable (few or no gold-substrate edges were included in images). The region absent in KBP-partial particles was now represented by clear density corresponding to an additional six helices and allowed modeling of the majority of KBP, excluding some longer disordered loops (Fig. 5 ▸ *e*). Under all sample-preparation conditions, successful processing of such a small particle (∼72 kDa) was highly dependent on ice thickness: additional data sets collected on grids without GO that exhibited thicker overall ice (Supplementary Fig. 3*a*) and therefore lacked the ice-thickness/particle-concentration gradient observed in optimal conditions (Figs. 1 ▸ *b* and 4 ▸ *c*) produced low-resolution 2D classes (secondary-structure elements not well resolved; Supplementary Fig. 3*b*) that were excluded from the final reconstruction.

### 3.4. A carbon grid support causes aggregation of KBP–kinesin complexes even with an overlaid graphene oxide substrate   

**Figure 6:**
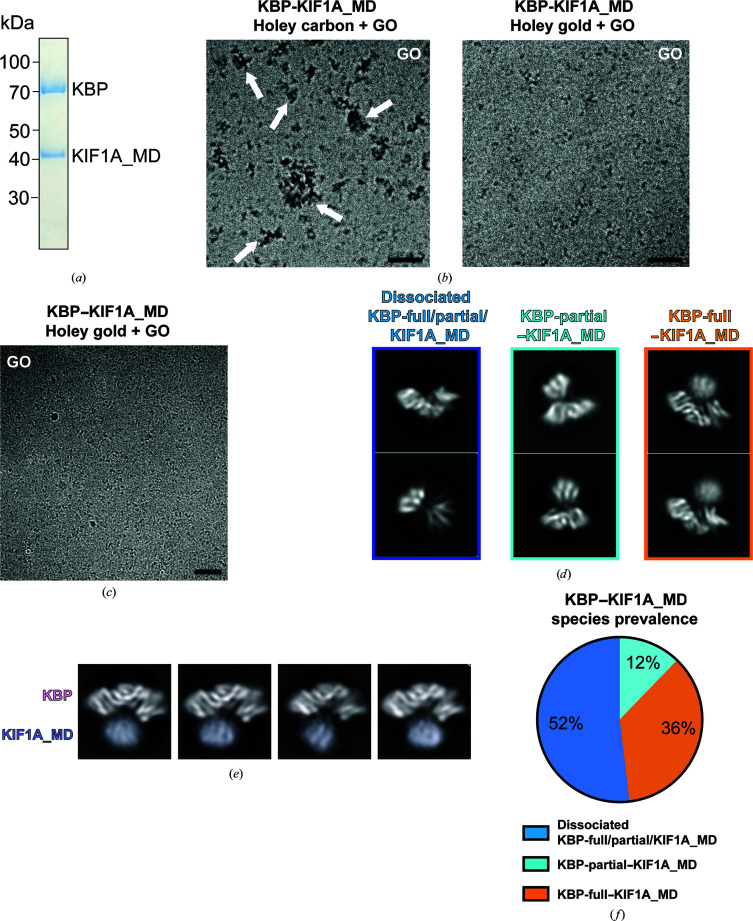
KBP–KIF1A_MD sample preparation on GO reveals heterogeneity and flexibility. (*a*) SDS–PAGE gel of purified KBP–KIF1A_MD complexes (∼110 kDa in total). (*b*) Representative screening micrographs (at 120 kV without a VPP; see Section 2) of KBP–KIF1A_MD complexes prepared on GO-coated holey carbon or gold grids, showing aggregation (white arrows) in the former case. GO, graphene oxide substrate. Scale bar = 40 nm. (*c*) Representative micrograph (data collection at 300 kV without a VPP) of KBP–KIF1A_MD complexes prepared on GO-coated holey gold grids. Scale bar = 40 nm. (*d*) Example 2D classes of dissociated KBP-full/partial/KIF1A_MD (left), KBP-partial–KIF1A_MD complexes (center) and KBP-full–KIF1A_MD complexes (right) derived from GO-coated holey gold grids. (*e*) Exemplar 2D classes of KBP-full–KIF1A_MD showing flexibility of the KIF1A_MD (blue false color) position and density blurring relative to KBP (pink false color), which has been roughly aligned between the different 2D classes. (*e*) Pie chart indicating the proportions of different species identified by 2D classification in the KBP–KIF1A_MD sample on holey gold plus GO grids.

To understand the mechanism of kinesin inhibition by KBP (Atherton *et al.*, 2020 ▸), we also studied KBP–kinesin MD complexes by cryo-EM. We targeted the MDs of two kinesins reported to bind KBP: the kinesin-3 KIF1A and the kinesin-12 KIF15 (see Section 2). We purified KBP–KIF1A_MD complexes (Fig. 6 ▸ *a*) and, with prior knowledge of the behavior of KBP during sample preparation, prepared KBP–KIF1A_MD complexes on grids with a GO substrate (Fig. 6 ▸ *b*). Surprisingly, preparation on GO-coated holey carbon grids caused moderate to severe aggregation of the sample but this was not observed when using GO-coated holey gold grids, despite otherwise identical preparation conditions (Fig. 6 ▸ *b*).

### 3.5. Two KBP–kinesin complexes exhibit different behavior on a graphene oxide substrate   

Data from GO-coated gold grids were collected at 300 kV on a Titan Krios with a K2 camera but without using a VPP: KBP–KIF1A_MD complexes are over 100 kDa and are therefore large enough to process successfully using standard defocus phase contrast (Fig. 6 ▸ *c*). 2D classification revealed a number of species, including dissociated KBP and likely KIF1A_MD subunits, KBP-partial associated with KIF1A_MD and KBP-full associated with KIF1A_MD (Fig. 6 ▸ *d*). Surprisingly, both KBP-full and KBP-partial associated with KIF1A_MD appeared to be flexible, presenting as either blurred KIF1A_MD density and/or a variable KIF1A_MD position relative to KBP in 2D classes (Figs. 6 ▸ *d* and 6 ▸ *e* and Supplementary Video S1). KBP-partial species associated with KIF1A_MD were rare (12% of particles, consistent with the use of GO preventing partial denaturation of KBP), while roughly half of the sample consisted of dissociated KBP or KIF1A_MD subunits (52% of particles) and roughly a third was KBP-full with flexibly associated KIF1A_MD (36% of particles; Fig. 6 ▸ *f*, Table 1 ▸). We also prepared KBP–KIF1A_MD complexes on holey gold grids without GO and collected a small preliminary data set to see whether more ordered KBP–KIF1A_MD complexes could be observed. However, 2D classification did not reveal any clear complexes, but instead indicated the presence of dissociated KBP (shapes/sizes suggesting mainly KBP-partial) and/or KIF1A_MD (Supplementary Figs. 4*a* and 4*b*, Table 1 ▸).

**Figure 7:**
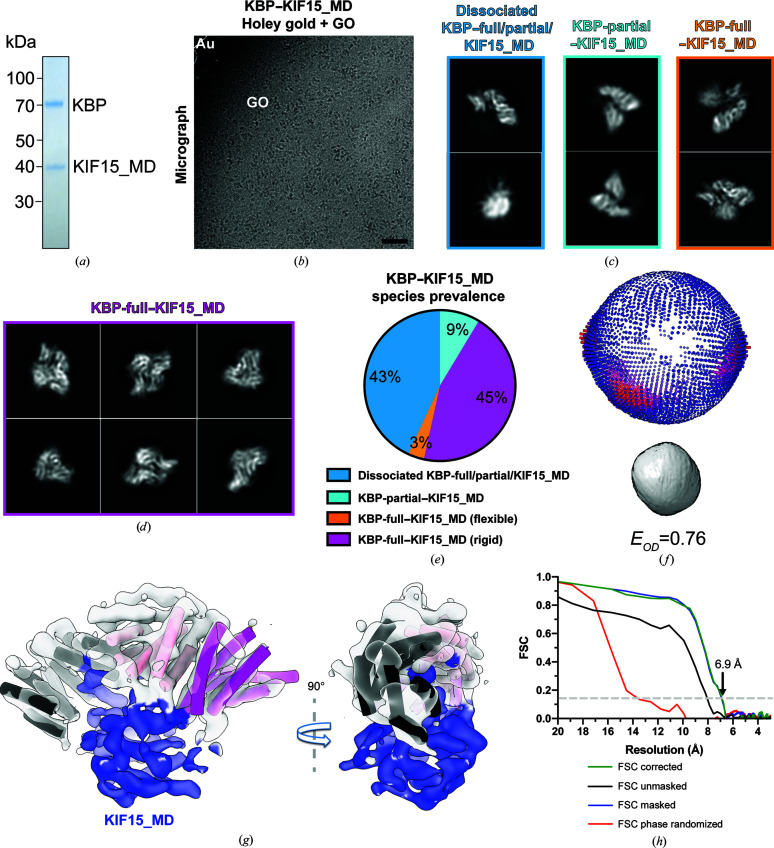
KBP–KIF15_MD6S sample preparation on GO leads to a 6.9 Å resolution 3D reconstruction from a subset of the data. (*a*) SDS–PAGE gel of purified KBP–KIF15_MD6S complexes (∼110 kDa in total). (*b*) Representative micrograph of KBP–KIF15_MD6S complexes prepared on GO-coated holey gold grids (data collection at 300 kV without a VPP). GO, graphene oxide substrate; Au, gold support. Scale bar = 40 nm. (*c*) Exemplar 2D classes of dissociated KBP-full/partial/KIF15_MD6S (left), KBP-partial–KIF15_MD6S complexes (center) and KBP-full–KIF15_MD6S complexes (right) derived from GO-coated holey gold grids. (*d*) Example 2D classes of KBP-full–KIF15_MD6S from GO-coated holey gold grids where the KIF15_MD6S position/conformation is rigid relative to KBP. (*e*) Pie chart indicating the proportions of different species identified by 2D classification in the KBP–KIF15_MD6S sample on holey gold plus GO grids. (*f*) 3D angular distribution (top, *RELION* output plot) and Fourier PSFs and *E*_od_ (below) for the KBP-full–KIF15_MD6S (rigid) data subset derived from both GO-coated holey gold grids. The orientation of the 3D angular distribution and Fourier PSF correspond to the KBP-full–KIF15_MD6S reconstruction orientation shown on the right in (*g*). An *E*_od_ of >0.7 indicates a good angular distribution (Naydenova & Russo, 2017 ▸). (*g*) 90° rotated views of the KBP-full–KIF15_MD6S (rigid) 3D reconstruction, with semi-transparent density for KBP in gray and KIF15_MD6S in blue. Helices of the fitted KBP model shown as tube representations and colored as in Figs. 1 ▸(*d*), 2 ▸(*f*) and 5 ▸(*e*). (*h*) Gold-standard Fourier shell correlation (FSC) curves between independent masked, unmasked, phase-randomized and corrected half-maps (Chen *et al.*, 2013 ▸) for the KBP-full–KIF15_MD6S (rigid) 3D reconstruction shown in (*g*), calculated by *RELION* version 3.1 (Zivanov *et al.*, 2018 ▸; 6.9 Å resolution at the ‘gold-standard’ 0.143 FSC cutoff).

Complexes of KBP with KIF15_MD6S (a cysteine-light KIF15_MD construct; see Section 2) were purified, samples were prepared on GO-coated gold EM grids and data were collected under similar conditions as for KBP–KIF1A_MD (Figs. 7 ▸ *a* and 7 ▸ *b*). As for KBP–KIF1A_MD complexes, a mixture of dissociated KBP and likely KIF15_MD6S subunits, KBP-partial associated with KIF15_MD6S and KBP-full associated with KIF15_MD6S were observed (Fig. 7 ▸ *c*). However, an additional subset of particles segregated into 2D classes of KBP-full–KIF15_MD6S complexes that showed clear secondary structure both in the KIF15_MD6S and KBP-full portions (Fig. 7 ▸ *d*). These ‘rigid’ complexes represented 45% of the data set, while KIF15 associated flexibly with KBP-full represented only 3% of the data set (Fig. 7 ▸ *e*, Table 1 ▸). The rigid KBP-full–KIF15_MD6S complexes provided a variety of 2D views of the complex, contributing to a good angular distribution and a relatively isotropic 3D reconstruction at 6.9 Å resolution (Figs. 7 ▸ *g* and 7 ▸ *h*, Table 1 ▸). Interestingly, KBP-full complexes with both flexibly attached KIF1A_MD and KIF15_MD6S adopted the same highly preferred orientations as did KBP alone on GO-coated grids (Supplementary Fig. S5).

## 4. Discussion   

Here, we have detailed our experiences in preparing KBP and KBP–kinesin MD complexes for cryo-EM, in particular relating to sample behavior on different EM grid types. The majority of KBP was found to be partially denatured when prepared in unsupported ice on holey carbon or gold grids, and we found that overlaying a GO substrate before sample application prevented this denaturation. As particles adsorbed to overlaid graphene substrates are sequestered from the air–water interface (D’Imprima *et al.*, 2019 ▸), it is likely that KBP adherence to GO prevents air–water interface interactions. In an analysis of a range of macromolecular cryo-EM samples, ∼90% of those tested adsorb to the air–water interface (Noble, Dandey *et al.*, 2018 ▸), leading to a number of effects, including denaturation of the particles (Glaeser & Han, 2017 ▸). Evidence suggests that the first particles reach the air–water interface rapidly, depending on the sample (Kudryashova *et al.*, 2005 ▸; Noble, Wei *et al.*, 2018 ▸; Taylor & Glaeser, 2008 ▸). A recent study with three benchmark samples suggested that while the majority of particles reach the air–water interface in <6 ms, they then undergo a slower equilibration process (taking up to a second) to settle into preferred orientations and/or undergo denaturation (Klebl *et al.*, 2020 ▸). Therefore, our observations suggest that the majority of KBP particles experienced partial denaturation after equilibration at the air–water interface during the delay between sample application to grids and vitrification. It is possible that the minority of KBP particles that were fully ordered in samples prepared without GO were located away from the air–water interface at the point of freezing due to either mechanical forces or some protective surface film of denatured protein (Noble, Dandey *et al.*, 2018 ▸). It should be noted, however, that we cannot discount the possibility that rather than denaturing upon contact with the air–water interface, KBP rests in a partially disordered state when soluble (away from the air–water interface) and is only fully stabilized by GO interaction. The generation of partially denatured species was recently observed due to the air–water interface in a sample of a much larger particle, fatty acid synthase, by cryo-EM and the authors reported that a hydrophilized graphene layer prevented this denaturation (D’Imprima *et al.*, 2019 ▸), much like we report here with a GO layer. Apart from graphene layers, detergents can be used in some circumstances as a method of preventing air–water interface interactions (Chen *et al.*, 2019 ▸; Glaeser & Han, 2017 ▸), although this is sample-dependent and the reduction in contrast due to background detergent may be prohibitive for smaller particles such as KBP.

We also observed effects arising from sample interaction with carbon or GO surfaces. Firstly, KBP particles adhered to GO exhibited strong preferential orientations. While sample tilting can be used to alleviate this issue in some cases, it is limited by difficulties in accurate CTF determination, resolution loss due to beam-induced motion and reduced signal to noise because of increased sample thickness in tilted grids (Brilot *et al.*, 2012 ▸; Lyumkis, 2019 ▸; Tan *et al.*, 2017 ▸). In the case of low-signal small particles this is particularly problematic, and it was prohibitive in the case of KBP. Detergent use without GO can also improve angular distributions by preventing preferred orientations at the air–water interface, but again may be problematic with small particles by reducing the contrast difference between the target particles and the surrounding buffer (Drulyte *et al.*, 2018 ▸). Secondly, KBP–kinesin complexes were observed to aggregate on GO-coated carbon grids but not on GO-coated gold grids. Variable and likely single-sided GO coverage means that a proportion of the sample, including that migrating to both sides of the grid during application or locating to spaces between carbon and GO layers, still interacts with the carbon surface. In this case, interaction of the sample with carbon possibly causes its aggregation, a proportion of which then washes onto the GO found over holes in the carbon. Many samples have a strong preference for carbon surfaces, while gold, being more inert than carbon, may reduce detrimental interactions between the grid surface and sample. Alternatively, unknown solubilized contaminants displaced from carbon-coated copper grids (but not gold grids) during sample application could interact with the sample, causing it to aggregate. Interestingly, we also noted subsets of KBP–KIF1A_MD and, to a lesser extent, KBP–KIF15_MD6S complexes where the kinesin_MD was flexibly associated with KBP. In these subsets, the KBP subunit within the complexes adopted strong preferential orientations on GO that were very similar to those adopted by KBP alone. Mutational analysis found that KBP interacts with KIF15 and KIF1A MDs in a similar manner (Atherton *et al.*, 2020 ▸). Therefore, the flexible complex subsets may represent sample-preparation artifacts on GO-coated grids to which KBP–KIF1A_MD6S is more susceptible.

New technologies are emerging that rapidly freeze samples on timescales that prevent some of the sample from reaching and/or equilibrating at the air–water interface, in some cases improving angular distributions and reducing particle denaturation (Dandey *et al.*, 2018 ▸, 2020 ▸; Jain *et al.*, 2012 ▸; Klebl *et al.*, 2020 ▸; Kontziampasis *et al.*, 2019 ▸; Noble, Wei *et al.*, 2018 ▸; Ravelli *et al.*, 2020 ▸). While under development to accelerate vitrification times, these technologies may prove advantageous over the use of detergents and graphene surfaces, which do not eliminate all surface interactions within the sample. Strategies for resolving small particles (<100 kDa) by cryo-EM are emerging. While we use a VPP here for the ∼70 kDa KBP, at present their use reduces data-collection efficiencies and limits resolution (Buijsse *et al.*, 2020 ▸). More information per unit electron damage and higher contrast in thin ice can be achieved at lower EM voltages (Peet *et al.*, 2019 ▸). Data collection at 200 kV rather than the standard 300 kV has already proved effective in solving small particle structures to near-atomic resolutions (Herzik *et al.*, 2017 ▸, 2019 ▸). Furthermore, EM hardware is currently under development for optimized data collection at 100 kV, with exciting results for small particles (Naydenova *et al.*, 2019 ▸). Nonetheless, new generations of direct electron detectors optimized for operation at 300 kV (such as K3 from Gatan or Falcon 4 from Thermo Fisher) offer advantages including larger fields of view, faster frame rates and improved detector quantum efficiencies (DQEs), generally aiding high-resolution macromolecular structural determination. Finally, minimizing the ice thickness (reducing solvent electron scattering events and increasing contrast) is of particular importance for successful structure determination of small macromolecules (<100 kDa), as was found in this work. On this note, it is emerging that particular EM grid types, hole sizes, support films (including graphene monolayers) and rapid vitrification methods tend to reduce ice thickness and flatten ice-thickness gradients, and can produce single rather than multiple overlapping layers of sample (Han *et al.*, 2020 ▸; Noble, Dandey *et al.*, 2018 ▸). The ongoing development of sample-preparation techniques that aim to utilize or avoid the effects of particle interactions with surfaces, along with the optimization of data-collection strategies and hardware, promises to revolutionize the structural determination of even very small macromolecules by cryo-EM.

## Supplementary Material

Supplementary Figures and full caption to Supplementary Video S1. DOI: 10.1107/S2059798321001935/id5010sup1.pdf

Supplementary Video S1. Flexible position of KIF1A_MD relative to KBP on GO. DOI: 10.1107/S2059798321001935/id5010sup2.mp4

## Figures and Tables

**Table 1:** Summary of preparation methods and sample observations

Preparation	KBP	KBP–KIF1A_MD	KBP–KIF15_MD6S
Holey carbon grids†	77% of particles partially denatured	Not tested	Not tested
	Good KBP-full angular distribution (*E*_OD_ = 0.86‡)		
Holey gold grids§	80% of particles partially denatured	Complex subunits dissociate	Not tested
	Good KBP-full angular distribution (*E*_OD_ = 0.86‡)		
Holey carbon grids† + GO	Only 6% of particles partially denatured	High level of sample aggregation	Not tested
	Poor KBP-full angular distribution (*E*_OD_ = 0.61‡)		
Holey carbon grids† + GO + 40° tilt	Poor 2D classes	Not tested	Not tested
Holey gold grids§ + GO	Not tested	52% of particles dissociated complex subunits	43% of particles dissociated complex subunits
		36% of particles a complex of KBP-full with flexible KIF1A_MD	3% of particles a complex of KBP-full with flexible KIF15_MD6S
		12% of particles a complex of KBP-partial with KIF1A_MD	9% of particles a complex of KBP-partial with KIF15_MD6S
			45% of particles a complex of KBP-full with KIF15_MD6S
			Good KBP-full–KIF15_MD6S angular distribution (*E*_OD_ = 0.76‡)

† C-flat 2/2 holey carbon EM grids (Protochips, Morrisville, North Carolina, USA).

‡ *E*_od_ is a statistical measure characterizing the angular distribution, where a value of 1 is a perfectly isotropic angular distribution and a value of ∼0.6 is suboptimal (Naydenova & Russo, 2017 ▸).

§ 1.2/1.3 UltrAuFoil gold EM grids (Quantifoil).
