# Supplementary material for: Cryo-EM of kinesin-binding protein: challenges and opportunities from protein-surface interactions
Source: Acta Crystallogr D Struct Biol. 2021 Mar 30;77(Pt 4):411–23. doi: 10.1107/S2059798321001935 (PMC8025885; doi:10.1107/S2059798321001935)
Supplement: Supplementary file 1 [file d-77-00411-sup1.pdf]

STRUCTURAL
BIOLOGY

Volume 77 (2021)

Supporting information for article:

Cryo-EM of kinesin-binding protein: challenges and opportunities from protein-surface interactions

Joseph Atherton and Carolyn A. Moores

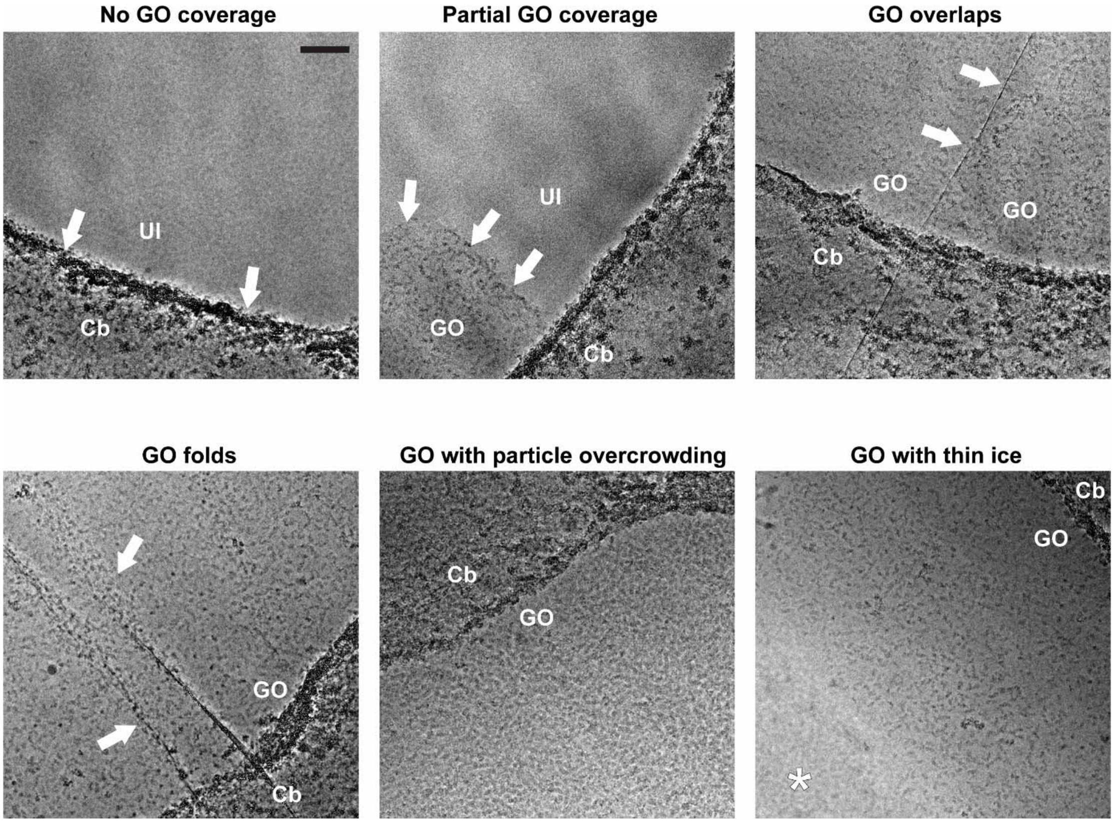

Figure S1 Examples of heterogeneity in GO coverage and KBP sample quality when prepared on GO-coated grids. Representative cryo-micrographs of KBP prepared on GO-coated holey carbon grids. Top-left; Example of a hole in the carbon without GO coverage. White arrows indicate particles adhered to the edge of the carbon hole, suggesting a strong preference for carbon surfaces. Top-centre; Example of a hole in the carbon with partial GO coverage. White arrows indicate the edge of the GO substrate. Top-right; Example of a hole in the carbon with overlapping (multi-layered) GO coverage. Note increased protein aggregation on one side of the overlap. Bottom-left; Example of a hole in the carbon with distorted GO coverage (folding). Note increased protein aggregation at folds (white arrows). Bottom-centre; Example of a GO-coated hole in the carbon with unusually high sample density for unknown reasons. Bottom-right; Example of a GO-coated hole in the carbon with an ice and protein concentration gradient. The area indicated with a white asterisk is possibly dry. Scale bar = 40 nm. GO = Graphene oxide layered over holes in the carbon. Cb = carbon support. UI = unsupported ice.

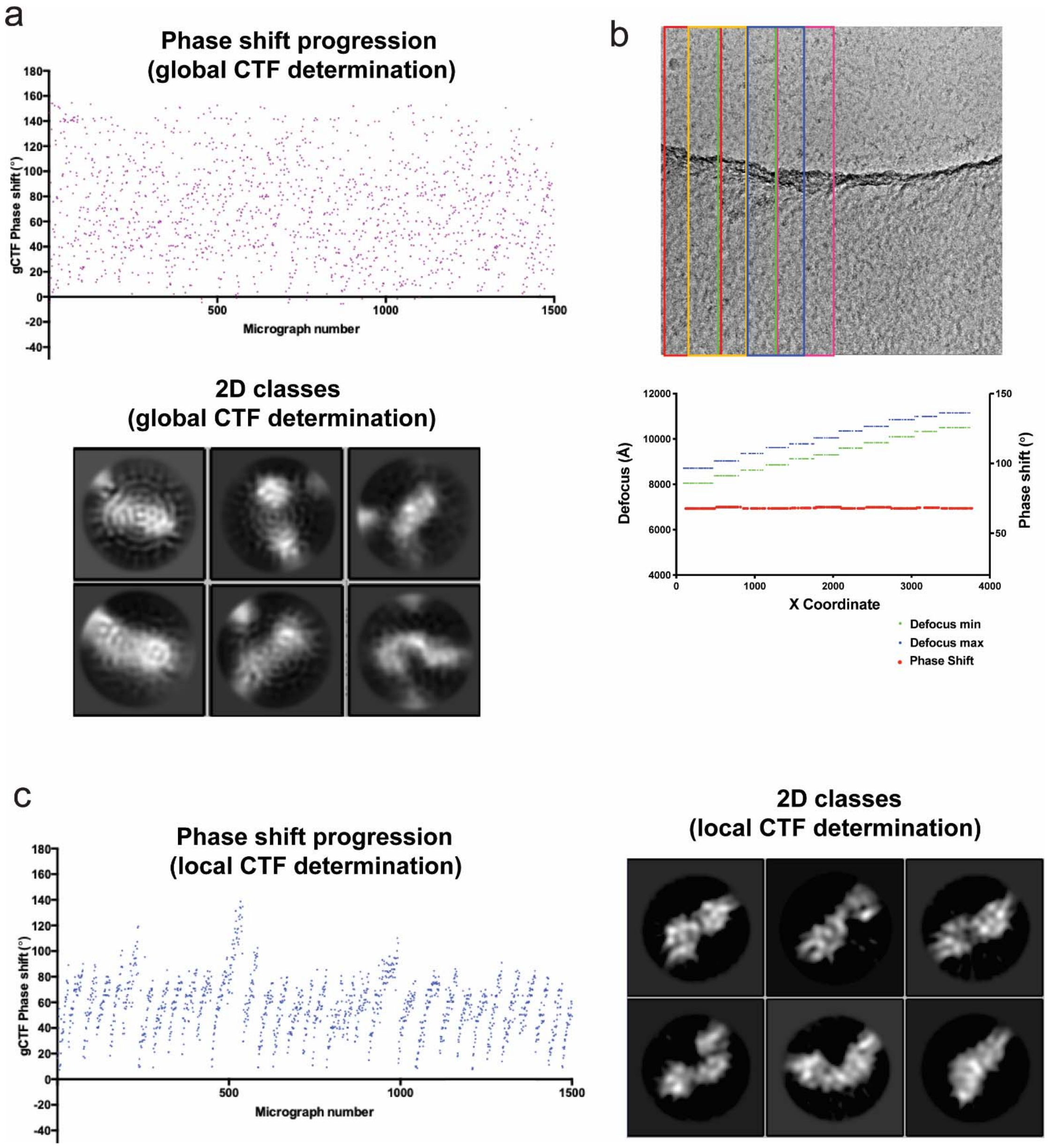

Figure S2 CTF determination in tilted VPP data. a) Top: Phase shifts calculated with gCTF for micrographs using standard global CTF determination across the whole tilted micrograph. The phase shifts do not show the expected progressions at new positions on the phase plate every 50 micrographs, as expected from the data collection parameters and indicative of poor CTF determination. Bottom: Exemplar well-populated 2D classes of KBP prepared on holey carbon grids coated with GO with 40° stage tilt applied, using standard global CTF determination. b) Top; Representative micrograph of KBP complexes prepared on GO-coated holey carbon grids at 40° tilt. Coloured boxes A-E represent overlapping strips perpendicular to the tilt-axis used to locally calculate CTF. Bottom; local gCTF defocus measurements and phase-shifts are calculated from these measurements perpendicular to the tilt axis (X-axis) and then assigned to each particle (each point on the graph represents a value for an individual particle). As expected defocus changes perpendicular to the tilt axis while the

calculated phase shifts remain essentially constant). Top: As for panel a, but using custom local strip-based CTF determination. With custom strip-based CTF determination, the phase-shifts show the expected progressions at new positions on the phase plate every 50 micrographs, indicating accurate CTF determination. Bottom: Exemplar well-populated 2D classes of KBP prepared on holey carbon grids coated with GO with 40° stage tilt applied, using custom local CTF determination.

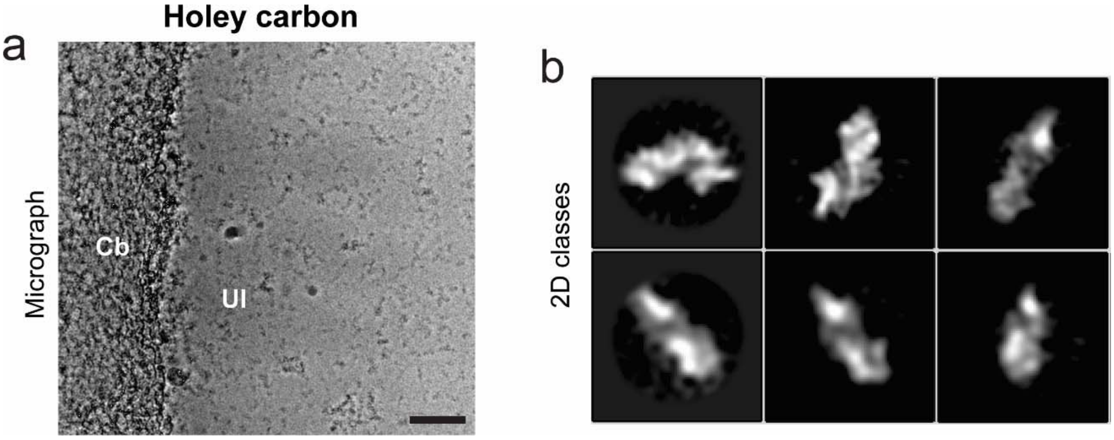

Figure S3 KBP data quality is highly sensitive to ice thickness. a) Representative micrograph of KBP prepared on holey carbon grids, exhibiting thicker ice in holes. In thicker ice conditions, the image lacks a clear gradient of ice thickness and protein concentration in holes in the carbon as observed in thinner ice conditions (e.g Fig. 1b). Cb = carbon support, UI = unsupported ice. Scale bar = 40 nm. b) Example rare 2D classes of KBP-full prepared on holey carbon grids exhibiting thicker ice. Note poorer quality 2D classes lacking clear secondary structure features (compare with Fig. 4b).

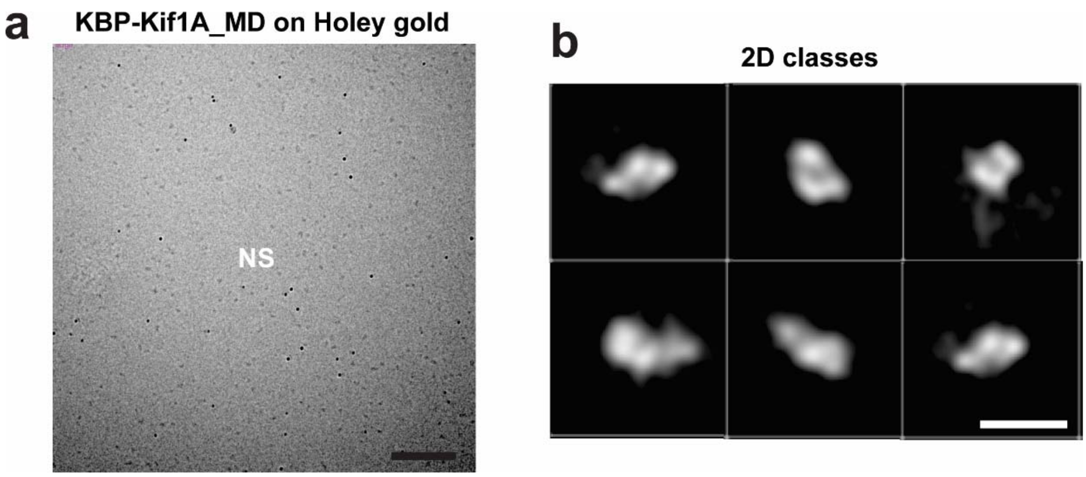

Figure S4 KBP-kinesin data quality is highly sensitive to ice thickness. a) Representative micrograph of KBP-KIF1A_MD prepared on holey gold grids without GO. Cb = carbon support, UI = unsupported ice. Scale bar = 40 nm. b) Example 2D classes from the preliminary KBP-KIF1A_MD dataset on holey gold grids without GO. 2D classes are of a size and shape suggesting few complexes remain. Scale bar = 8 nm

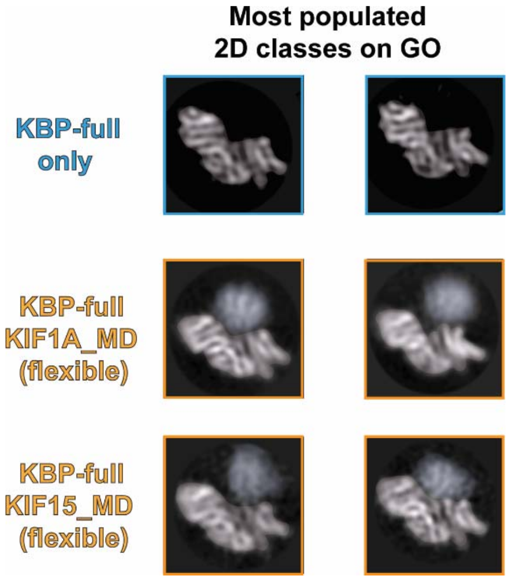

Figure S5 Comparison of highly preferred orientations on GO in KBP-only preparations and flexible complex subgroups in KBP-kinesin_MD preparations. The most well-populated 2D classes prepared on GO of; upper panels, KBP alone, middle panels, KBP-full-KIF1A_MD showing flexibility of KIF1A_MD, lower panels, KBP-full-KIF15_MD6S showing flexibility of KIF15_MD6S. 2D in-plane KBP orientation has been roughly aligned between the different 2D classes. KBP is overlaid with pink false colour, while kinesin_MDs are overlaid with blue false colour.

Supplementary Video S1. Flexible position of KIF1A_MD relative to KBP on GO. 2D classes exhibiting similar views of KBP were roughly aligned upon the KBP density portion (top). A movie though the 2D classes clearly shows movement of KIF1A_MD density (bottom) relative to KBP indicating a flexible position.
